# Soluplus Graft Copolymer: Potential Novel Carrier Polymer in Electrospinning of Nanofibrous Drug Delivery Systems for Wound Therapy

**DOI:** 10.1155/2014/789765

**Published:** 2014-01-20

**Authors:** Urve Paaver, Ingrid Tamm, Ivo Laidmäe, Andres Lust, Kalle Kirsimäe, Peep Veski, Karin Kogermann, Jyrki Heinämäki

**Affiliations:** ^1^Department of Pharmacy, Faculty of Medicine, University of Tartu, Nooruse 1, 50411 Tartu, Estonia; ^2^Immunology Group, Institute of Biomedicine and Translational Medicine, Faculty of Medicine, University of Tartu, Ravila 19, 50411 Tartu, Estonia; ^3^Institute of Ecology and Earth Sciences, University of Tartu, Ravila 14a, 50411 Tartu, Estonia

## Abstract

Electrospinning is an effective method in preparing polymeric nanofibrous drug delivery systems (DDSs) for topical wound healing and skin burn therapy applications. The aim of the present study was to investigate a new synthetic graft copolymer (Soluplus) as a hydrophilic carrier polymer in electrospinning of nanofibrous DDSs. Soluplus (polyvinyl caprolactam-polyvinyl acetate-polyethylene glycol graft copolymer (PCL-PVAc-PEG)) was applied in the nonwoven nanomats loaded with piroxicam (PRX) as a poorly water-soluble drug. Raman spectroscopy, X-ray powder diffraction, differential scanning calorimetry, and scanning electron microscopy (SEM) were used in the physical characterization of nanofibrous DDSs. According to the SEM results, the drug-loaded PCL-PVAc-PEG nanofibers were circular in cross-section with an average diameter ranging from 500 nm up to 2 **µ**m. Electrospinning stabilized the amorphous state of PRX. In addition, consistent and sustained-release profile was achieved with the present nanofibrous DDSs at the physiologically relevant temperature and pH applicable in wound healing therapy. In conclusion, electrospinning can be used to prepare nanofibrous DDSs of PCL-PVAc-PEG graft copolymer (Soluplus) and to stabilize the amorphous state of a poorly water-soluble PRX. The use of this synthetic graft copolymer can open new options to formulate nanofibrous DDSs for wound healing.

## 1. Introduction

Modern polymeric drug delivery systems (DDSs) for wound therapy are systems which are designed to release drug(s) to wound sites in a consistent and sustained fashion [1, 2]. In recent years, electrospun polymeric nonwoven nanomats have found multiple uses as DDSs in wound healing and skin burn therapy [3–7]. Electrospinning is the method of choice for preparing polymeric nanofibrous DDSs with unique characteristics including ultrafine structure, a large surface area to volume ratio, and a high porosity with a small pore size [8–12]. The working principle of electrospinning is relatively simple: a polymer solution is ejected from a capillary toward a grounded metal collector plate by applying high voltage between the capillary and the plate [13]. Established polymers, such as cellulose derivatives, polyvinylpyrrolidone, polyvinyl alcohol, poly-L-lactic acid, poly(**ε**-caprolactone), and chitosan, have been applied as carriers in the electrospinning of nanomats for drug delivery applications. The selection of a carrier polymer for electrospinning is crucial since the type of polymer and drug-polymer-solvent interactions influence the formation, morphology, mechanical properties, drug release (including burst effect), and biocompatibility of the final nanofibrous DDSs [10, 12, 14–18]. Among current nanofabrication methods, electrospinning is considered the process with potential for industrial-scale mass production [19].

**Figure 1:**
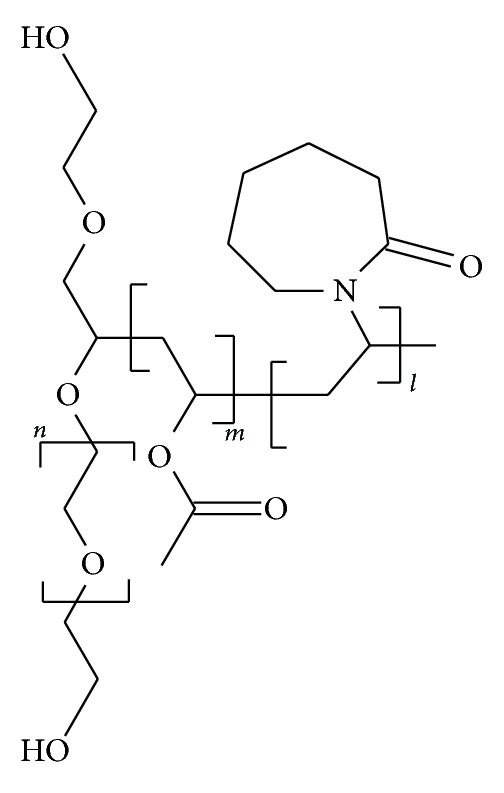
Chemical structure of Soluplus (polyvinyl caprolactam-polyvinyl acetate-polyethylene glycol graft copolymer (PCL-PVAc-PEG)).

Soluplus (polyvinyl caprolactam-polyvinyl acetate-polyethylene glycol graft copolymer (PCL-PVAc-PEG)) (Figure 1) is a new pharmaceutical excipient designed originally for preparing solid solutions of poorly water-soluble drugs by hot-melt extrusion technology [20]. Soluplus is a water-soluble copolymer with the average molecular weight ranging from 90,000 to 140,000 g/mol, and it is capable of solubilizing poorly water-soluble drugs [21]. To date, there is only little information available about the use of Soluplus in electrospinning and other nanofabrication-based methods. Recently, Nagy et al. [22] compared electrospun and extruded Soluplus-based oral solid dosage forms for improving the dissolution of a poorly water-soluble drug. To our knowledge, Soluplus has not been applied as a carrier polymer in electrospun drug-loaded polymeric nanofibers and nanofibrous mats designed as topical DDSs for wound healing and burn therapy. A water-soluble carrier polymer allows aqueous-based environment-friendly electrospinning process and water-soluble drug loadings in the topical nanomats. In addition, porous hydrophilic nanofibrous membranes have been shown to provide better wound healing performance than hydrophobic ones [23].

In this study, Soluplus (PCL-PVAc-PEG graft copolymer) was investigated as a potential novel water-soluble carrier polymer in electrospinning. Furthermore, a strong hypothesis was that the present graft copolymer can be used as a carrier polymer in the aqueous-based electrospinning of nonwoven nanofibrous DDSs for wound healing. In the present study, Soluplus was used in the nanomats loaded with piroxicam (PRX) as a model drug. Raman spectroscopy, X-ray powder diffraction (XRPD), differential scanning calorimetry (DSC), and scanning electron microscopy (SEM) were applied for the physical characterization of nanofibrous DDSs.

## 2. Materials and Methods

### 2.1. Materials

Soluplus (polyvinyl caprolactam-polyvinyl acetate-polyethylene glycol graft copolymer (PCL-PVAc-PEG)) was kindly gifted from BASF SE Pharma Ingredients & Services, Ludwigshafen, Germany. Piroxicam (anhydrous PRX pure form I, PRXAH I, Letco Medical, Inc., USA) was used as a model drug when preparing electrospun nanomats. Acetone (Sigma-Aldrich C.C.) was applied as solvent in electrospinning. Physical mixtures of PRX and PCL-PVAc-PEG were prepared at a ratio of 1 : 13 (w/w) to study the effects of electrospinning on the solid-state properties of the materials.

### 2.2. Electrospinning of Nanofibrous DDSs

**Figure 2:**
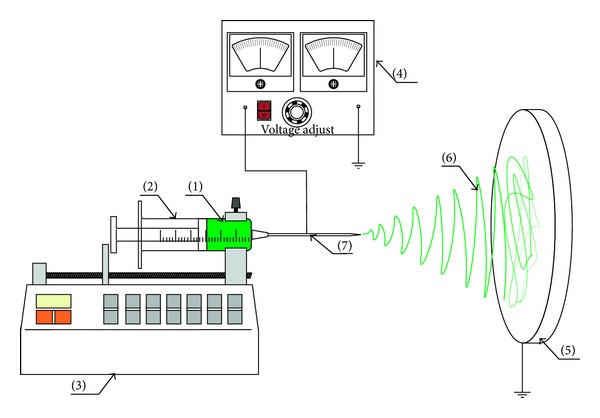
Electrospinning setup applied for preparing drug-loaded nanofibers and nonwoven nanomats. (1) Polymer solution, (2) syringe, (3) syringe pump, (4) high-voltage power supply, (5) grounded collector, (6) polymer mesh (nanofibrous mat), and (7) electrode.

The electrospinning setup and experimental conditions used to prepare the nanofibrous DDSs are shown in Figure 2. An automatic syringe pump KD Scientific (Model number: KDS-250-CE, Geneq Inc., USA) provided a feed rate of 2 mL/h. The high-voltage power supply Gamma High Voltage Research (Model number ES3OP-10W/DAM, USA) generated the DC voltage of 9 kV. The size of the needle used was 23 G. The distance between the spinneret and the fiber collector plate was 10 cm. The ratio of PRX and PCL-PVAc-PEG used in the nanofibers was 1 : 13 (w/w), and the total polymer concentration in acetone was 33% w/v. The final theoretical concentration of PRX in the electrospun nanofibers was 7.5% (w/w).

### 2.3. Scanning Electron Microscopy

A high-resolution scanning electron microscope (SEM) (Zeiss EVO 15 MA, Germany) was used to investigate the diameter of fibers and surface morphology of nanofibrous DDSs. Samples were mounted on aluminum stubs with silver paint and were magnetron-sputter coated with a 3 nm gold layer in an argon atmosphere prior to microscopy imaging.

### 2.4. X-Ray Powder Diffraction (XRPD)

X-ray diffraction patterns of the starting materials and electrospun nanofibers were studied with a X-ray diffractometer (D8 Advance, Bruker AXS GmbH, Germany). The XRPD experiments were carried out in symmetrical reflection mode (Bragg-Brentano geometry) with CuK_*α*_ radiation (1.54 Å). The scattered intensities were measured with the LynxEye one-dimensional detector including 165 channels. The angular range was from 5° to 30° 2-theta with the step size of 0.2° 2-theta. The total measuring time was 498 s/step. The operating current and voltage were 40 mA and 40 kV, respectively. Experimental results on nanofibers were compared to the theoretical patterns in the Cambridge Structural Database (CSD, Cambridge, UK). Ref codes BIYSEH [24] and BIYSEH02 [25] were used as reference crystal structures for PRXAH I and PRX form II (PRXAH II), respectively.

### 2.5. Raman Spectroscopy

Raman spectra on both starting materials, their physical mixtures, and electrospun nanofibers were collected using a Raman spectrometer equipped with a thermoelectrically cooled CCD detector (1024 × 64) and a fiber optic probe (B&W Tek Inc., USA). A 300 mW laser source at 785 nm was used (B&W Tek Inc., USA). Spectra were recorded between 200 and 2200 cm^−1^ with an integration time of 12 s, and each spectrum was the average of three scans. B&W Tek software (B&W Tek Inc., Newark, DE, USA) was used for the collection of Raman spectra.

### 2.6. Differential Scanning Calorimetry (DSC)

Thermal properties of the starting materials, their physical mixtures, and produced nanofibers were measured using a differential scanning calorimeter (DSC 4000, Perkin Elmer Ltd., Shelton, CT, USA). The DSC system was calibrated for temperature and enthalpy using indium as a standard. Samples of 2-3 mg were sealed in an aluminium pan. In case of pure PRX, the sample size of 0.2 mg was used to be comparable with the other samples tested. A nitrogen purge with a flow rate of 20 mL/min was used in the furnace. The scans were obtained by heating from 30°C to 220°C at a rate of 20°C/min. Each run was performed in triplicate.

### 2.7. *In Vitro* Drug Release

The *in vitro* drug release experiments were carried out in an automated dissolution system (Termostat-Sotax AT7, Sotax AG, Switzerland) equipped with rotating baskets (USP XXVIII, Apparatus 2) [26]. An internal standard was prepared by dissolving 20 mg of PRXAH in 0.02 M sodium hydroxide solution. The sample size of the nanofibers was 140 ± 3 mg (containing theoretically 10 mg of PRX). The concentration of PRX in the dissolution medium (900 mL) was measured at 354 nm by using a UV-VIS spectrophotometer (Ultrospec III, Biochrom Ltd., UK). Since PRX has two pKa values (pKa 1.86 and 5.46) and the different forms of the molecules have different solubility and dissolution in the release media, it was important to perform *in vitro* dissolution tests in more than one pH [27, 28]. Sink-conditions were maintained at all time in the *in vitro* dissolution tests. The dissolution media used were purified water, the USP XXVIII phosphate buffer solution pH 7.2, and the USP XXVIII buffer solution pH 1.2 at 37 ± 0.5°C. The pH of the buffer solutions was confirmed by the pH meter HI 9024 (Hanna Instruments, USA). The basket rotation speed was set at 50 rpm. The samples were collected using an automated sampling and filtering system at 3 min intervals over a period of 2 hours. Five parallel tests were performed for each nanofiber system.

## 3. Results and Discussion

According to Boateng et al. [1], the use of hydrophilic polymers as medicated wound dressings has great promise because of the potential advantages they offer. Such advantages include, for example, drug delivery to wound sites in a consistent fashion, avoidance of high systemic doses, and patient compliance in chronic wound management. These polymers are also easily washed off from the wound surface. In the present study, a new hydrophilic synthetic PCL-PVAc-PEG graft copolymer (Soluplus) was studied as a carrier polymer in the electrospun nanofibrous DDSs (nonwoven nanomats) intended for wound healing applications. Synthetic polymers are preferred (over natural polymers) for electrospinning since they are strong, cheap, and reliable and exert physicochemical characteristics that can be controlled through the production process [29].

### 3.1. Electrospinning of PVAc-PEG Graft Copolymer (Soluplus)

The major bottle neck for using new carrier polymers in electrospun nanofibers is a limited knowledge of their behavior in electrospinning, selection of a proper solvent system, and the physical solid-state properties of the materials in the final nanomats. Moreover, electrospinning process conditions (i.e., relative humidity and temperature) can affect the structure and mechanical properties of the electrospun nanofibers and alter their applications [30]. In the present study, the method development involved the solubility screening tests of drug and copolymer (data not shown) and subsequent electrospinning trials to find an electrospinnable solvent system and optimal process parameters, respectively. The drug loading of nanofibers was successfully made by electrospinning of drug/copolymer blends 1 : 13 (w/w) and using the total copolymer concentration of 33% w/v in acetone. Acetone was selected since a quick solvent evaporation is essential for the formation of homogeneous nanofibrous mats. In comparison to other electrospinnable polymers described in the literature, Soluplus could enable fabrication of “greener” nanofibers for pharmaceutical DDSs through aqueous-based (or less toxic solvent-based) processing. To date, frequently used solvents in electrospinning of polymeric DDSs are, for example, dichloromethane, hexafluoroisopropanol, trifluoroacetic acid, and trifluoroethylene [31].

### 3.2. Physical Appearance, Fiber Size, and Surface Morphology

**Figure 3:**
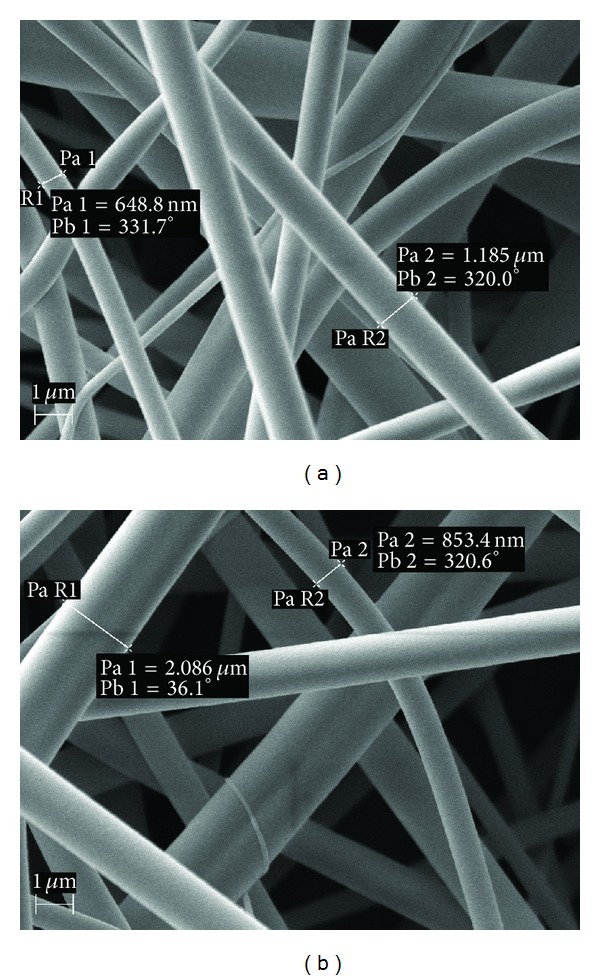
Representative SEM micrographs of the electrospun nanofibers of PCL-PVAc-PEG graft copolymer (Soluplus) loaded with a model drug, piroxicam (PRX). (a) SEM micrograph showing the nanomat with 3D layered fiber mesh structure, nonwoven pattern, and the absence of beads; (b) SEM micrograph showing some individual nanofibers with a fiber thickness over 2 *μ*m. Magnification: 20,000x.

The drug-loaded nanomats of PCL-PVAc-PEG graft copolymer (Soluplus) exhibited a 3D layered fiber mesh structure, nonwoven pattern, and the absence of beads. According to SEM shown in Figure 3, the nanofibers were circular in cross-section with an average diameter ranging from 500 nm up to 2 *μ*m. The nanofibers were yellow in color suggesting that PRX existed in amorphous form [32, 33]. The amorphous form of drug exhibits random position of molecules relative to another, shows short-range order over a few molecular dimensions, and has physical properties (including solubility) quite different from those of the corresponding crystalline state [34]. The presence of amorphous PRX in the nanofibers was confirmed by XRPD and Raman spectroscopy (data shown in the following section). The absence of beads (= equals the processing defects) suggested the appropriate solvent and process parameter selection for the present medicated nanofibrous system. According to the literature, the beads are readily formed in electrospinning if the surface tension of a solution (solvent system) is high or if the viscosity of a solution is low [15]. Readers are referred to two excellent review articles for details on the effects of polymeric solutions on electrospinning [29, 35].

### 3.3. Solid-State and Thermal Properties

**Figure 4:**
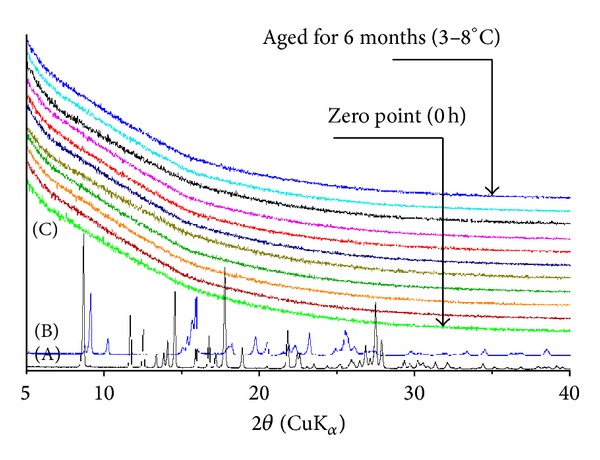
XRPD patterns of crystalline anhydrous piroxicam (PRX) forms and electrospun nanofibers of PCL-PVAc-PEG graft copolymer (Soluplus) loaded with PRX. Theoretical reference patterns of PRX anhydrous forms I (PRXAH I, BIYSEH) and II (PRXAH II, BIYSEH02) obtained from the Cambridge Structural Database (CSD, Cambridge, UK). (A) PRXAH I; (B) PRXAH II; and (C) fresh and aged drug-loaded nanofibers of PRX and graft copolymer (Soluplus) (1 : 13 w/w).

**Figure 5:**
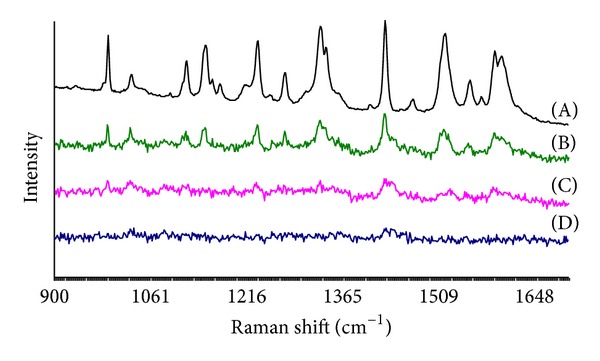
Raman spectra of physical mixture and electrospun nanofibers of piroxicam (PRX) anhydrous form I (PRXAH I) and PCL-PVAc-PEG graft copolymer (Soluplus). (A) Pure PRXAH I; (B) physical mixture of PRXAH I and PCL-PVAc-PEG graft copolymer (Soluplus) (1 : 13 w/w); (C) PCL-PVAc-PEG graft copolymer (Soluplus) nanofibers loaded with PRXAH I in a 1 : 13 w/w drug-polymer ratio; (D) pure PCL-PVAc-PEG graft copolymer (Soluplus) nanofibers.

Application of electrospinning and hydrophilic carrier polymer(s) could be an interesting approach for the stabilization of poorly water-soluble drugs in amorphous state, and consequently, modifying their delivery rate to the site of action in wound therapy. Since the drug is simply dissolved in the polymer solution prior to electrospinning, the formation of an amorphous drug is evident during the process [29]. The amorphous form of drug exhibits an enhanced solubility compared to the crystalline state, but being physically unstable it has a tendency to spontaneously recrystallize [34]. In the present study, electrospinning was found to be the method to form and stabilize the amorphous state of PRX when preparing the nanofibrous DDSs of PRX and PCL-PVAc-PEG graft copolymer. XRPD results revealed that the nanofibers contained PRX in amorphous form immediately after fabrication and after a six-month aging period, when stored at low temperature (3–8°C) and humidity (RH 0%) (Figure 4). Thus, these results were also verified by Raman spectroscopy (Figure 5).

The electrospun nanofibers showed different Raman spectra compared to their respective physical mixture. With the physical mixture of PRX and carrier polymer (1 : 13 w/w), the characteristic Raman spectroscopy peaks for PRXAH I were displayed at 1282, 1338, 1435, and 1525 cm^−1^ (Figure 5(A)). With the electrospun nanofibers, the Raman spectroscopy peaks for the crystalline PRXAH forms disappeared and the presence of the characteristic peaks for amorphous PRX was observed at 1237, 1477, 1531, and 1561 cm^−1^ (Figure 5(B)). These characteristic peaks for amorphous PRX (peak position, intensity, and the sharpness of the peaks) differ from all known PRX crystalline forms. In electrospinning, a large specific surface area of nanofibers results in a fast and efficient evaporation of solvent, and consequently, molecules have no time to (re)crystallize and hence they most likely obtain an amorphous supermolecular structure [14, 36].

**Figure 6:**
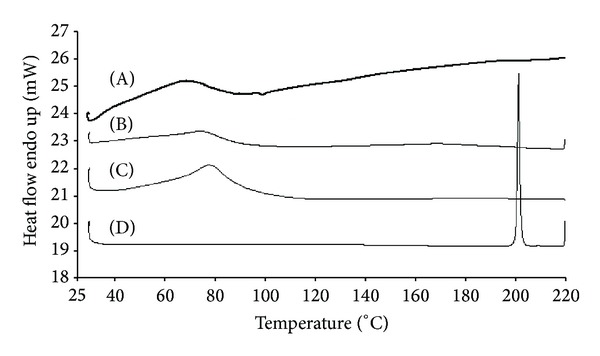
DSC thermograms of the starting materials, physical mixture, and electrospun nanofibers of PCL-PVAc-PEG graft copolymer (Soluplus) loaded with PRX. (A) Drug-loaded nanofibers of PRX and graft copolymer (Soluplus) (1 : 13 w/w); (B) physical mixture of PRX anhydrous form I (PRXAH I) and graft copolymer (Soluplus) (1 : 13 w/w); (C) PCL-PVAc-PEG graft copolymer (Soluplus); (D) pure PRXAH I.

DSC thermographs (Figure 6) confirmed the presence of PRX in amorphous form in the electrospun nanofibrous mats. No melting endotherm was observed at the expected melting point range of PRXAH I at approximately 199–201°C. In the literature, the crystallization of drugs in electrospun polymeric nanofibers has been related to a phase separation and salt formation even with only a 1-2% (w/w) of drug loading [7, 36, 37]. As shown in Figure 6, both PCL-PVAc-PEG graft copolymer (Soluplus) and PRX-loaded polymeric nanofibers presented a broad dehydration endotherm in a range of 30°C–110°C, which was attributed to the removal of water adsorbed by hydrophilic carrier polymer. This broad dehydration endotherm also overlapped with the characteristic glass transition temperature, *T*_*g*_ (70°C) of a pure PCL-PVAc-PEG graft copolymer (Soluplus) [20]. Interestingly, the PRX-loaded polymeric nanofibers exhibited the second (even wider) endotherm ranging from 100°C to 220°C, presumably suggesting that a physical interaction or fusion of PRX into the PCL-PVAc-PEG graft copolymer (Soluplus) had occurred. Recently, Lust et al. [38] reported that the dehydration endotherm of amorphous solid dispersions of PRX and PCL-PVAc-PEG graft copolymer (Soluplus) was narrower than that of the respective physical mixtures. The latter was attributed to the increased temperature used to prepare the solid dispersions by melting method [38]. They also showed a broad endotherm ranging from 125°C to 185°C with the physical mixtures of PRX and the present carrier polymer, but in contrast to the present results on nanofibers, Lust et al. did not observe an endotherm at higher temperatures with the amorphous solid dispersions prepared by melting method.

### 3.4. Drug Release *In Vitro*

**Figure 7:**
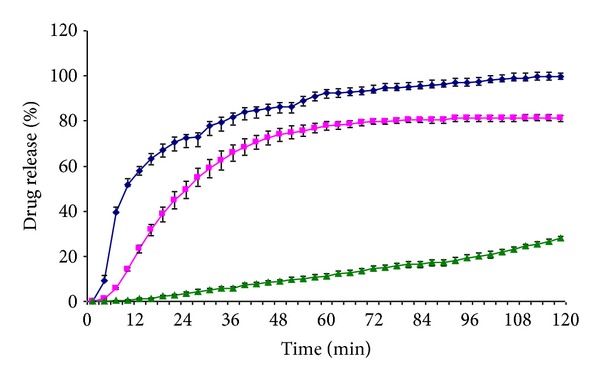
*In vitro* drug release of the electrospun nonwoven nanomats of PRX and PCL-PVAc-PEG graft copolymer (1 : 13 w/w ratio) in purified water (purple square), pH 7.2 phosphate buffer (green triangle), and pH 1.2 buffer solution (blue diamond) at 37 ± 0.5°C (*n* = 5).

The ideal modern wound dressing should provide both immediate (anesthetic) drug delivery for pain alleviation and sustained (antibiotic) activity for prophylaxis against bacterial infections [7]. In the present study, the electrospun nanofibrous DDSs of PRX and PCL-PVAc-PEG graft copolymer provided drug delivery ranging from two to several hours and exhibited an absence of lag time in the dissolution test *in vitro* (Figure 7). It is evident that the amorphous state of PRX in the nanofibers leads to a relatively short lag time for the drug release *in vitro*. The *in vitro* drug release profiles of the present nanofibers were found to be also pH and ionic-strength dependent. As shown in Figure 7, the drug release rate was significantly decreased when the pH of a dissolution medium was increased from pH 1.2 to pH 7.2, exhibiting a sustained fashion drug release profile at the pH close to the physiological pH of the skin. The burst release of drug (in the first hour) followed by a plateau was observed at lower pH 1.2, and the drug release rate of PRX was slightly decreased when the dissolution medium was changed to purified water. These findings are in agreement with a recent study by Hughey et al. [39]. Hughey et al. investigated the effects of kosmotropic salts (anions) on the gel strength and solubility behavior of Soluplus and stated that at the physiologically relevant temperature of 37°C, the cloud point of Soluplus was reached in deionized water and phosphate buffer (pH 6.8) but not in 0.1 N hydrochloric acid (pH 1.2) [39]. This most likely explains the prolonged drug release profile of the nanofibrous DDSs obtained in the present study at pH 7.2 (phosphate buffer solution). The dissolution of nanofibers at pH 7.2 (i.e., close to skin pH), however, showed zero-order kinetics and a clear increase in the total time of drug release (Figure 7). This result is in agreement with the behavior of a water-soluble polymer or of a low-molecular-weight polymer, and suggests an erosion-controlled drug release mechanism from the present nanofibrous mats.

## 4. Conclusions

Electrospinning can be used to prepare nanofibrous DDSs based on a new synthetic PCL-PVAc-PEG graft copolymer (Soluplus) with potential application in wound therapy. Moreover, electrospinning and the present copolymer stabilize the amorphous state of PRX. No significant solid-state incompatibilities are expected in the electrospun binary mixtures of PRX and the present synthetic graft copolymer. Consistent and sustained-release fashion drug delivery was achieved at the physiologically relevant temperature and pH regions applicable in wound healing and burn therapy. This sustained-release fashion drug release can be useful in medicated wound dressings for a long-term pain relief or prophylaxis against bacterial infections. The drug release behavior of the present nanofibers are pH and ionic-strength dependent due to the solubility behavior of PCL-PVAc-PEG graft copolymer (Soluplus). Further studies will be needed on the applicability and performance of these nanofibrous DDSs in wound healing and burn therapy applications.
